# An Appraisal of Pumpkin Seed Extract in 1, 2-Dimethylhydrazine Induced Colon Cancer in Wistar Rats

**DOI:** 10.1155/2018/6086490

**Published:** 2018-09-02

**Authors:** K. Yogeswara Chari, Picheswara Rao Polu, Rekha R. Shenoy

**Affiliations:** ^1^Department of Pharmacology, Manipal College of Pharmaceutical Sciences, Manipal Academy of Higher Education, Manipal 576104, Karnataka, India; ^2^Department of Pharmacognosy, Manipal College of Pharmaceutical Sciences, Manipal Academy of Higher Education, Manipal 576104, Karnataka, India

## Abstract

**Background:**

Cancer is one of the most important public health burdens in developed and developing countries. Colon cancer (CC) is the sixth most common cause of death in India and third most important cause in developed countries. For treating cancer, several synthetic agents are available but they cause side effects. Therefore, there is a need to investigate plant derived anticancer agents with lesser side effects. In this direction, we have made an attempt to unravel the potential of pumpkin seed extract for treating colon cancer.

**Objective:**

The objective of this study was to evaluate pumpkin seed extract as prophylactic and treatment for 1, 2-dimethylhydrazine (DMH) induced colon cancer in Wistar rats.

**Materials and Methods:**

Male Wistar rats were divided into seven groups, namely, control, DMH (disease control), 5-Flurouracil (standard), treatment groups (100mg/kg and 200 mg/kg), and pretreatment groups (100mg/kg and 200 mg/kg) with pumpkin seed extract. The animals were euthanised at the end of study and colons were examined.

**Results:**

A significant difference in the aberrant crypt foci (ACF) number in all treatment groups compared to control and DMH groups were noted. Pretreatment group at a dose of 200 mg/kg showed a significant decrease in the colon length/weight ratio. Pretreatment groups showed a significant change in the colonic glutathione (GSH) and superoxide dismutase (SOD) levels when compared to control and DMH control. The nitrite content was decreased in treatment group 200 mg/kg at 5.203±0.852 when compared to DMH control at 8.506±3.866. All treatment groups demonstrated decreased hyperplasia and ACF in histology.

**Conclusion:**

Pumpkin seed may prevent the risk of CC when consumed in dietary proportions.

## 1. Introduction

Plants have been pertinent to humans as medicinal sources and many modern drugs have been derived from secondary plant metabolites. Since time immemorial, mankind has been dependent on plants for their curative properties. Across the world, a major source of food comes from plants. Most of the vegetables are eaten along with seeds; however, there are a few in which the seeds are discarded such as pumpkin, sapota, and apple etc. Seeds play a pivotal role in germination. In other words, they are life giving. One such is the pumpkin seed [PS], which is removed during cooking. Pumpkin or* Cucurbita pepo* is native to Northern Mexico and southwestern and eastern USA. It is an annual climber with five angled stems and grows up to 15 m long. Roots are branched and grow in a well-developed taproot. Fruits vary in shape, color, and size. The flesh of the fruits is in different colors like green, yellow, and white. The shapes of the fruits are oval, flattened, cylindrical, globular, fusiform, and scalloped. The leaves are simple, alternate, and broadly ovate to deltoid, palmately lobed, 20-30 cm long and 10–35 cm leaves with 5-25cm petioles. Pumpkin is a monoecious plant, which produces nectar from its actinomorphic flowers [1]. Several therapeutic activities like antioxidant [2], hepatoprotective [3], hypolipidaemic [3], antidiabetic [4], wound healing [5], anti-inflammatory [6], antibacterial [7], suppressing stimulated mononuclear cells [8], antihypertensive [9], antiarthritis [10], antidepressant activity [11], anticancer [12], antihyperlipidaemic [13], and anticataract [14] have been reported. Interestingly, the seeds of pumpkin have been reported to show cytotoxic activity in specific cancer cell lines* in vitro* [12]. However, PS which was tested in HCT-116 cell lines has not been investigated* in vivo* in experimental model of colon cancer earlier.

Colon cancer is the sixth most common cause of death in India and third important cause in developed countries. In 2017, as indicated by the American Cancer Society, there will be an expected 95,520 new instances of colon malignancy, in which 47,700 will be in men and 47,820 in women. 40% deaths from rectal malignancy are misclassified as colon tumour [15]. Despite the advanced treatment for CRC, which comprises 5‐Fluorouracil, oxaliplatin, and EGFR, VEGFR inhibitors, the absolute burden of CRC has increased in India, with unsuccessful treatments and limitations of the existing therapy.

The earliest hallmark for colon cancer is aberrant crypt foci (ACF). These are enlarged, aberrant, and elliptical shape crypts, which can be distinguished by comparing with control group. Among animal models of colon cancer, the use of chemical inducing agents such as 1, 2-dimethylhydrazine (DMH) at a dose of 20 mg/kg to male Wistar rats is considered as a well-established model. DMH is a highly specific colon carcinogen and is metabolically activated in the liver through a series of intermediates like azomethane, azoxymethane, and methylazoxymethanol acetate (MAMA). MAMA is conjugated with glucuronic acid in liver and excreted in bile. It enters directly into epithelial cells of the colon from blood circulation, where MAMA produces reactive oxygen species and methyl diazonium ions, responsible for the methylation of DNA and modification of nucleic acids and histones. Aberrant crypt foci can be observed within two weeks of DMH administration [16]. The objective of the present study was to evaluate pumpkin seed extract as prophylactic and treatment for 1, 2-dimethylhydrazine (DMH) induced colon cancer in Wistar rats. Hence, the study was proposed under the hypothesis that PS could attenuate colon cancer* in vivo.*

## 2. Materials and Methods

### 2.1. Reagents and Chemicals

1, 2-Dimethylhydrazine (DMH) was purchased from TCI chemicals, Chennai, India. Methylene blue was purchased from Himedia Laboratories, Mumbai, India. All the solvents and chemicals used were procured from Himedia Laboratories, Invitrogen, and Sigma-Aldrich Chemicals Private Ltd.

### 2.2. Plant Material

The seeds of* Cucurbita pepo* were collected in September 2017 from Manipal, Karnataka, India. The identity of the species was confirmed by Dr. Usharani S Suvarna, Head, Department of Botany, Mahatma Gandhi Memorial College, Udupi, Karnataka, and authenticated under the registration number MGMC/2018-19.

### 2.3. Preparation of Pumpkin Seed Extract

Approximately, 900 g of dried pumpkin seeds was collected and coarsely powdered with the help of mortar and pestle.

The extraction apparatus was constructed by using stands and clamps for supporting the Soxhlet and condenser. 2.5 litres of 80% ethanol was taken in a round bottomed flask, placed on an isomantle. The Soxhlet extractor and condenser were attached to isomantle. Coarsely powdered dried pumpkin seeds were placed into the thimble present inside the Soxhlet extractor. Glass wool was placed in the side arm to prevent the entry of solid particles. Ethanol in the round bottomed flask was heated using the isomantle at boiling point of 78°C. Solvent evaporated and moved through the side arm of Soxhlet extractor to the condenser. The condenser was continuously supplied with cold water. The evaporated solvent condensed and fell as drops into the thimble. This was a continuous process until it reached the siphon tube. Then the solvent present in siphon tube poured back into the round bottomed flask and the cycle began again. Standard time for running this process was a total of 36 hours.

Once the procedure was completed, a rotavapor was used to recover 80% ethanol, leaving a little quantity of extract in the round bottomed flask [17].

### 2.4. Phytochemical Investigation [18]

#### 2.4.1. Qualitative Test

This test was used to identify the active constituents present in the extract of* Cucurbita pepo. *The following tests were performed for the analysis of the crude extract.

#### 2.4.2. Liebermann-Burchard's Test

Solution was prepared using 2 mg of dry pumpkin seed extract in acetic anhydride, then it was boiled and cooled. After cooling, concentrated sulphuric acid (1ml) was added slowly to the sides of the test tube, until a brown ring formed at the junction of two layers. Formation of green color indicated that steroids were present.

### 2.5. Salkowski Test

2ml chloroform was added to the test solution, followed by few drops of concentrated sulphuric acid, and shaken well. Formation of reddish brown color at bottom layer indicated the presence of triterpenoids.

### 2.6. Test for Phytosterol

Acetic anhydride (2ml) was added to the extract. 1 or 2 drops of concentrated sulphuric acid was added to the above solution slowly through the sides of test tube. The presence of phytosterol was identified by an array of color change.

### 2.7. Test for Alkaloids

#### 2.7.1. Picric Acid Test

1gm of picric acid was weighed and dissolved in 100 ml of water. Appropriate quantity of pumpkin seed extract was added to the above solution. Creamy precipitate formed was an indication of presence of alkaloids.

#### 2.7.2. Test for Flavonoids

The pumpkin seed extract was filtered with water in a test tube and 5 ml of ammonia solution was added. Then few drops of concentrated sulphuric acid were added through the sides of test tube. Presence of flavonoids was identified by yellow color formation in the test tube. The yellow color disappeared after sometime.

### 2.8. Test for Tannins

2.5gm of pumpkin seed extract and distilled water (5ml) were added to the test tube and filtered. To the filtrate, suitable amount of ferric chloride dissolved in distilled water was added. The presence of tannins was identified by the formation of blue-black, green, or blue-green precipitate.

### 2.9. Test for Saponins

Distilled water (20ml) was used to dilute the pumpkin seed extract. The suspension was allowed to shake in a test tube. The formation of foam up to 2 cm indicated the presence of saponins.

### 2.10. Test for Carbohydrates

#### 2.10.1. Benedict's Test

0.5ml of Benedict's reagent was added to 0.5ml of filtrate. The mixture was heated for 2 minutes on hot water bath. Presence of carbohydrates was indicated by the formation of red color precipitate.

#### 2.10.2. Quantitative Test [18]

*Estimation of Total Phenolic Content*. Gallic acid is the most commonly used standard to determine total phenolic content. Standard stock solution of 100 *μ*g/ml was prepared. Different concentrations of standard were prepared ranging from 1-10 *μ*g/ml. Solution of the extract was prepared by dissolving 1 mg in 1 ml of distilled water. 100 *μ*l of extract solution was taken in a test tube and 0.75ml of reagent was added. Standard solutions were also mixed with the reagent and all the solutions were incubated for 5 min. 0.75ml of 6% sodium carbonate was added and incubated at room temperature for 90 min. Absorbance was measured at 725 nm using UV spectrophotometer.

*Estimation of Total Flavonoid Content*. Total flavonoid content was determined by using aluminium chloride colorimetric method. The extract solution was prepared by dissolving 1 mg in 1 ml of distilled water. Extract solution (0.5ml), methanol (1.5 ml), and 1 M potassium acetate (0.1 ml) were added to the test tubes. The volume of solution in test tubes was made up with distilled water to 5 ml. Then the solution was allowed to incubate at room temperature for 20 mins. The absorbance was measured at 415 nm after incubation. Standard calibration plot was determined by using quercetin (standard).

*Estimation of Total Tannin Content*. Tannic acid is a commonly used standard to determine total tannin content. Concentrations ranging from 1 to 10 *μ*g/ml of standard and 10 *μ*g/ml extract solution were prepared. 100-*μ*l standard/extract sample, 9.3 ml of water, and 300 *μ*l of Folin-Denis reagent were added and incubated in dark for 1 hour. Absorbance was measured at 760 nm. Standard graph was plotted. The total tannin content present in the extract was determined and expressed in terms of tannic acid equivalents.

*Estimation of Saponins*. 20 g of pumpkin seed extract was weighed and taken in a beaker. To the beaker, 200 ml of 20% ethanol was added. The beaker was kept on a water bath and temperature was maintained at 55°C and continuously stirred for 4 h. The resulting solution present in the beaker was collected and filtered by Whatman filter paper. The residue was again reextracted by adding 200 ml of 20% ethanol. The process was repeated by heating on the water bath at 55°C and stirred continuously for 4 h. The obtained extracts from first and second extraction were combined and kept on water bath at 90°C. Further, the beaker was kept on water bath until volume of solution reduced to 40 ml. The concentrated solution was taken in a separating funnel and 20 ml of diethyl ether was added and shaken vigorously. After shaking, diethyl ether layer and aqueous layer were separated in a separating funnel. The aqueous layer was collected by opening the knob and diethyl layer was discarded. The obtained aqueous layer was again washed with 60 ml of n-butanol and 10 ml of 5% aqueous sodium chloride was added. The n-butanol layer was collected and kept on a water bath for complete evaporation. The sample was allowed to dry completely in the oven into a constant weight. The percentage saponins was calculated using the following:

(1) $$\begin{array}{c} \text{Percentage saponins}=\frac{W2-W1}{\text{Weight of sample}}*100 \end{array}$$

W1 is weight of empty beaker, W2 is weight of empty beaker with sample.

### 2.11. Animal Procurement

Male Wistar rats weighing 180-200g were procured from Central Animal Research Facility, Manipal Academy of Higher Education, Manipal, Karnataka. Animals were acclimatized to the experimental room bearing the temperature 23±3°C, adequate humidity conditions, and 14:10 hours of light and dark cycle. The handling and caring of animals were done as per the guidelines put forth by the Institutional Animal Ethics Committee (IAEC), Manipal. The committee has cleared the study conducted and has issued the clearance certificate bearing the number, IAEC/KMC/45/2017.

### 2.12. Acute Toxicity Studies

We referred to OECD guideline no. 425 to perform acute oral toxicity. Female Wistar rats were procured and kept for overnight fasting before drug administration. PS extract was given as suspension in 0.25% carboxymethyl cellulose (CMC) and the dose was calculated based on body weight of Wistar rats. As per the OECD guidelines, three female Wistar rats received a dose of 2000 mg/kg. The animals were observed for a time period of 4, 8, 12, and 24 hrs for changes in the awareness, mood, CNS activity, muscle tone, reflexes, and autonomic profile. Further, the animals were kept under observation for 15 days for occurrence of any abnormalities.

### 2.13. Preparation of Carcinogen

1, 2-Dimethylhydrazine solution was prepared by using 1 mM EDTA and the pH was adjusted to 6.5 with 1 M sodium hydroxide. The induction phase was 20 weeks where the carcinogen was administered once weekly in a dose of 20 mg/kg (*i.p.*) for first 10 weeks and 30 mg/kg (*i.p.*) for the next 10 weeks [16].

### 2.14. Experimental Design

54 Male Wistar rats were used in this study. The rats were randomized and allocated into a group of six each. The pumpkin seed extract was tested as both treatment and pretreatment orally.

 
**Group I:** Control
 
**Group II:** Disease control (DMH: 20 mg/kg for first 10 weeks and 30 mg/kg for next 10 weeks, i.p.)
 
**Group III:** Disease + 5-Fluorouracil (10 mg/kg, i.p.)
 
**Group IV:** Disease + pumpkin seed extract (100mg/kg, p.o.)
 
**Group V:** Disease + pumpkin seed extract (200mg/kg, p.o.)
 
**Group VI:** Pumpkin seed extract (100 mg/kg, p.o.) + disease
 
**Group VII:** Pumpkin seed extract (200 mg/kg, p.o.) + disease

### 2.15. Experimental Procedure

After the induction of colon cancer, standard drug 5-Fluorouracil (10 mg/kg/*i.p.* weekly once) and pumpkin seed extracts (100 mg/kg and 200 mg/kg* p.o.* daily) were given.

In the pretreatment groups, rats were treated with only pumpkin seed extracts for an initial period of 8 weeks. After 8 weeks, DMH was administered weekly once for 20 weeks (20 mg/kg/*i.p.* for first 10 weeks and 30 mg/kg/*i.p.* for next 10 weeks) [19].

### 2.16. *In Vivo* Methods [19]

#### 2.16.1. Determination of ACF

After euthanasia of the experimental animals, the entire colon was dissected, opened in a longitudinal manner, and washed with saline. A piece of colon measuring 8 cm each was fixed on a filter paper in 10% buffered formalin for 24 hours. Then the colons were stained with 0.1% methylene blue in phosphate buffer saline for 20 min and aberrant crypt foci (ACF) counted. ACF number was determined by carefully examining the specimens under a compound light microscope at 40X magnification, with the mucosal side facing upwards. The stained ACF was identified by darker staining and large size, thick epithelial lining and elliptical luminal opening, elongated in length from lamina to basal surface of cells.

#### 2.16.2. Haematological Evaluation

Blood from anaesthetized animals was collected from the retro-orbital plexus before euthanasia. 50 *μ*l of 10% EDTA was used as an anti-coagulant for each 500 *μ*l of blood sample. The collected blood was analyzed through veterinary blood cell counter for hematological parameters.

### 2.17. Colon Length to Weight Ratio

Isolated colons were measured for its length in centimeters and weight in grams and the ratio was calculated as

(2) $$\begin{array}{c} \frac{\text{Colon length}}{\text{Weight ratio}}=\frac{\text{length in cm}}{\text{Weight in gram}} \end{array}$$

### 2.18. Spleen Index and Liver Index

Spleen and liver were collected and the index was calculated as

(3) Spleen or liver index=Weight of liver or spleen in gramsBody weight of animals in grams

### 2.19. ACF Incidence

ACF incidence indicated the percentage of animals with cancer induction out of all DMH treated animals. It was calculated as

(4) Percentage of ACF incidence=number of rats with ACFnumber of rats examined∗100

### 2.20. Biochemical Estimations

At the end of the study, blood was collected for biochemical parameters. After euthanasia, liver and colon were isolated from each animal following transcardial perfusion with ice-cold saline. The ice-cold potassium chloride (150mM) solution was used to prepare 10% tissue homogenate using a homogenizer (Remi, Mumbai, Maharashtra). The colon homogenate was used for estimations of glutathione (GSH), catalase [20], malondialdehyde (MDA) content or lipid peroxidation [21], nitrite, total protein [22], and superoxide dismutase (SOD) [23].

### 2.21. Histopathological Studies [24]

Dissected colon was placed in sample container filled with 10% formalin. Later, pieces of colon tissue were placed in 50 ml alcohol of different concentrations like 50%, 70%, 90%, and 100% for 5-7 hours. Further, the colonic sample was placed in xylene to obtain a clear and transparent tissue. Later, it was placed in a histology embedder consisting of four bowls filled with melted paraffin. The paraffin was distributed equally with the tissue. This process was carried at 54-60°C for 6-8 hours. The paraffin was allowed to cool at room temperature or by immersion of the block in cold water to solidify. The tissue blocks were placed in a microtome producing thin sections of same thickness. The optimal thickness for viewing under light microscope is 5–10 *μ*m. The thin sections of tissue were placed in hot water bath at 52°C to avoid the wrinkle formation. The thin section of tissues, which was placed in water bath, was transferred by dipping the slide into water bath. After drying, the slides were kept on hot plate to remove the paraffin wax present on the slide. The sections were stained with hematoxylin and eosin stain and observed under light microscope at 4, 10, and 40X magnification to capture the image.

### 2.22. Statistical Analysis

The results were represented as mean ± SEM and statistical analysis was carried out using one-way Analysis of Variance (ANOVA) followed by post hoc Tukey's test for comparison between groups, by Graph Pad Prism software version 5.0 (San Diego, CA, USA). p<0.05 was considered to be statistically significant.

## 3. Results

### 3.1. Phytochemical Investigation

#### 3.1.1. Qualitative Analysis

The pumpkin seed extract tested positive for flavonoids, triterpenoids, steroids, tannins, phytosterol, and saponins. Alkaloids and carbohydrates were absent as shown in Table 1.

#### 3.1.2. Quantitative Analysis

Quantitative analysis was performed to quantify the content of main constituents like flavonoids, saponins, phenols, and tannins.

*Estimation of Total Flavonoid Content*. Aluminium chloride method was mostly used to determine total flavanoid content and standard plot obtained using quercetin as standard. The total flavonoid content in the given sample was found to be 2.91 mg quercetin equivalents/g.

*Estimation of Total Phenolic Content*. The total phenolic content is expressed as mg gallic acid equivalents/g. The total phenolic content in pumpkin seed extract was found to be 3.85 mg GAE/g.

*Estimation of Tannin Content*. Tannic acid was used as standard for obtaining the standard plot. The total tannin content in pumpkin seed extract was found to be 0.814 mg TAE/g.

*Estimation of Total Saponins Content*. The percentage of total saponins content in the pumpkin seed extract was found to be 74.1%.

*Acute Toxicity Study*. In the acute oral toxicity study, there were no signs of toxic symptoms or mortality in female Wistar rats at a dose of 2000 mg/kg. Based on the results, pumpkin seed extract at a dose of 100 and 200 mg/kg was selected for the* in vivo* studies.

*Determination of ACF*. No ACF developed in control group which received vehicle. A significant increase in the number of ACF was observed in DMH control when compared to both control and standard. Both treatment and pretreatment groups, at doses 100 and 200 mg, were able to contain ACF significantly at p<0.05 as given in Table 2.

*Haematological Parameters*. PS extract at doses of 100 and 200 mg/kg (both preventive and curative), 5-Flurouracil (10 mg/kg), showed significant changes in WBC, lymphocytes, granulocytes, and monocytes when compared to DMH group as depicted in Table 3

**Figure 1:**
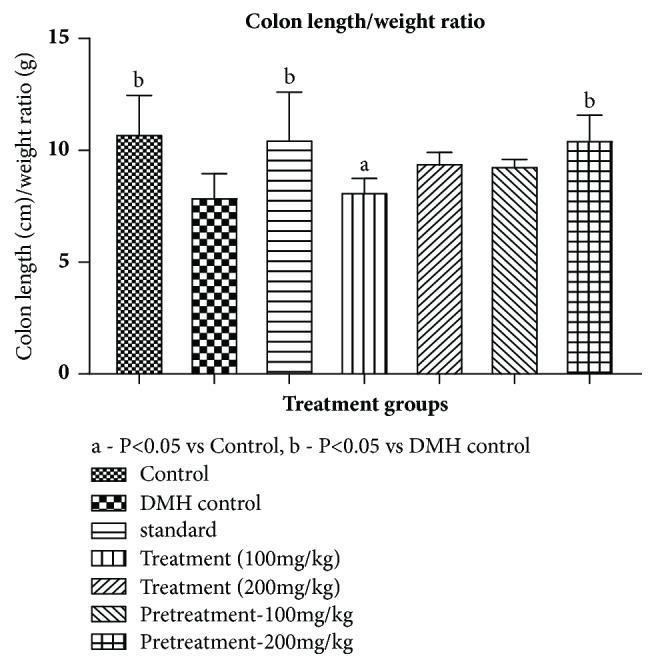
Effect of pumpkin seed extract on colon length/weight ratio. All values are mean ± SEM of six samples, ^a^P<0.05 versus Control, ^b^P<0.05 versus DMH control.

*Colon Length/Weight Ratio*. The formation of microadenomas, adenomas, and adenocarcinomas decreases the colon length/ weight ratio. There was a significant decrease in the colon length/weight ratio in DMH group as compared to control group. In the treatment groups, the ratio increased but was not significant with DMH control group. Pretreated group at 200 mg/kg showed significant increase in the ratios when compared to disease control as provided in Table 4 and Figure 1

*Spleen Index and Liver Index*. In spleen index, pretreatment at 200 mg/kg showed significant difference as compared to control group. When compared with DMH control, no treatment groups showed any significant difference. In liver index, both treatment and pretreatment at 200 mg/kg showed significant difference as compared to control group. The results are given in Table 5.

*Biochemical Estimations*

**Figure 2:**
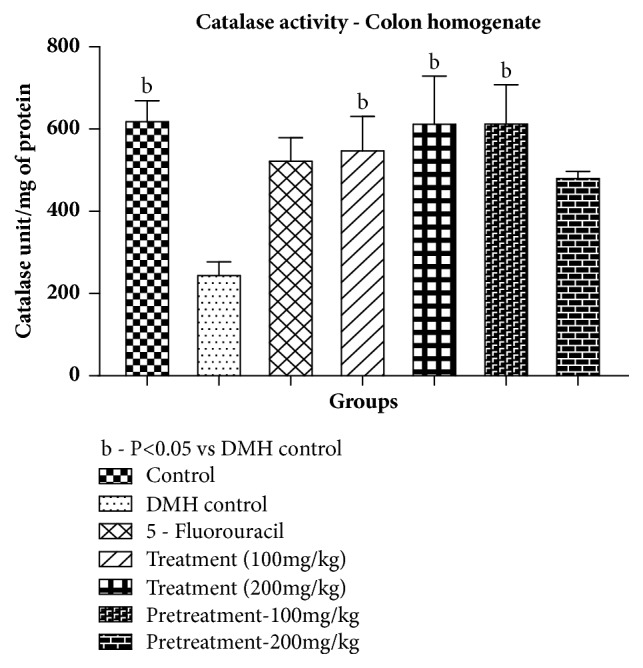
Effect of pumpkin seed extract on catalase. All values are mean ± SEM of six samples, ^b^P<0.05 versus DMH control.

*Catalase*. A significant decline in catalase levels of DMH group was found when compared to control group. On the other hand, treatments at both dose levels and pretreatment at 100 mg/kg showed significant (p<0.05) increase in catalase content when compared with DMH group as shown in Table 6 and Figure 2.

#### 3.1.3. Reduced Glutathione

**Figure 3:**
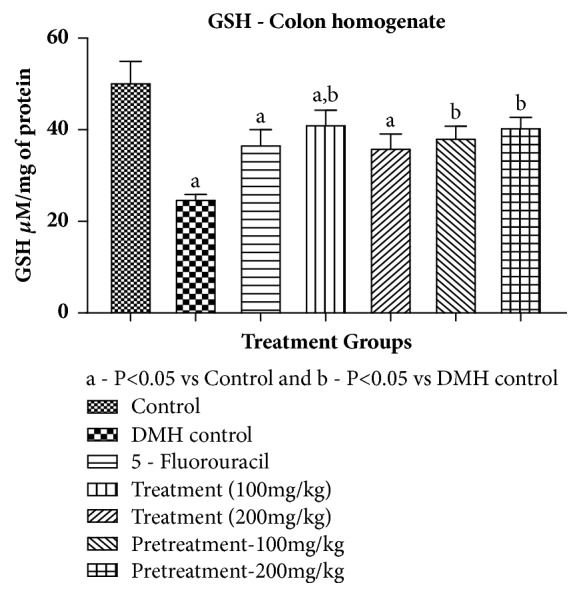
Effect of pumpkin seed extract on GSH. All values are mean ± SEM of six samples, ^a^P<0.05 versus Control, ^b^P<0.05 versus DMH control.

A significant decrease in GSH levels was noted in DMH treated rats as compared to control group. All treatments and 5-Fluorouracil groups showed significant increase in GSH content when compared to DMH group. The results are provided in Table 7 and Figure 3.

#### 3.1.4. Lipid Peroxidation or MDA Content

**Figure 4:**
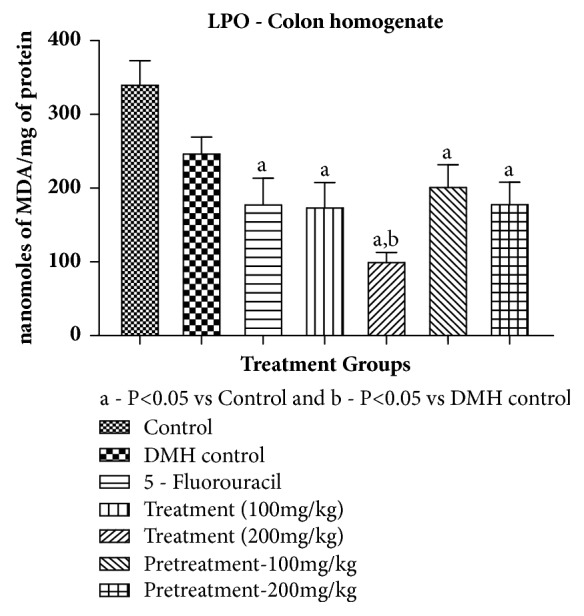
Effect of pumpkin seed extract on lipid peroxidation or MDA content. All values are mean ± SEM of six samples, ^a^P<0.05 versus Control, ^b^P<0.05 versus DMH Control.

It was observed that MDA content significantly increased in DMH administered rats, but was not significant when compared with control group. All the treatment groups and 5-Fluorouracil arm showed significant difference as compared to control. The treatment groups were able to reduce the MDA content as compared to DMH control but not significantly except treatment 200 mg/kg. The results were given in Table 8 and Figure 4.

**Figure 5:**
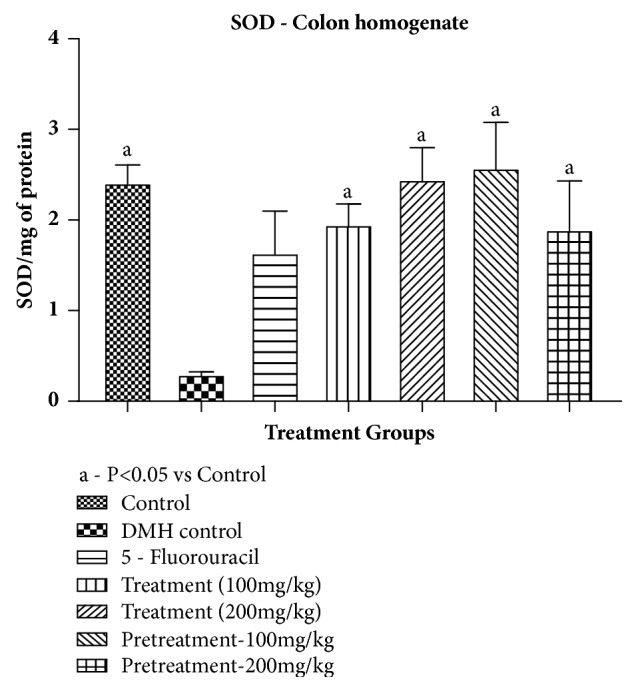
Effect of pumpkin seed extract on SOD. All values are mean ± SEM of six samples, ^a^P<0.05 versus DMH Control.

*Superoxide Dismutase*. SOD content was significantly decreased in DMH control when compared to control group. All the treatment groups showed significant increase in SOD content as compared to DMH control except 5-Flurouracil group as shown in Table 9 and Figure 5.

**Figure 6:**
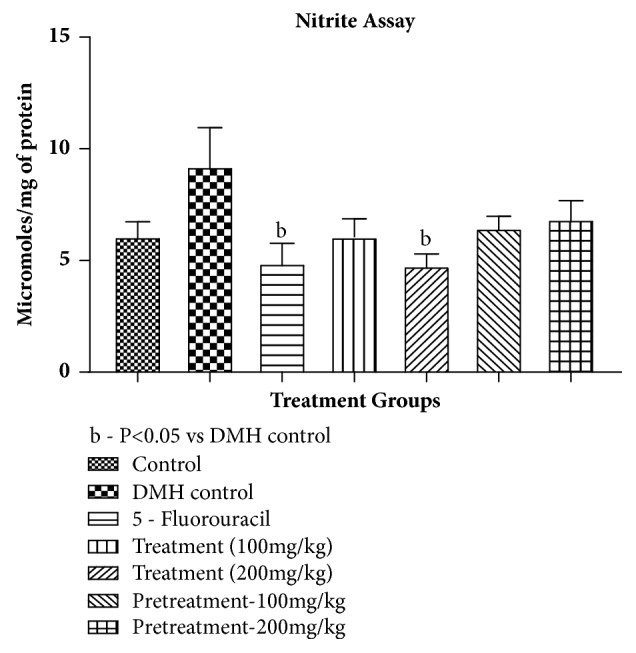
Effect of pumpkin seed extract on nitrite content. All values are mean ± SEM of six samples, ^b^P<0.05 versus DMH Control.

*Nitrite Content*. An increase in nitrite content was observed in DMH control group but was not significant when compared to control. Treatment group 200 mg/kg and 5-Fluorouracil significantly decreased the nitrite levels as compared to DMH control. Rest of the treatment groups decreased the nitrite levels but was not significant as compared to control and DMH treatment (given in Table 10 and Figure 6).

**Figure 7:**
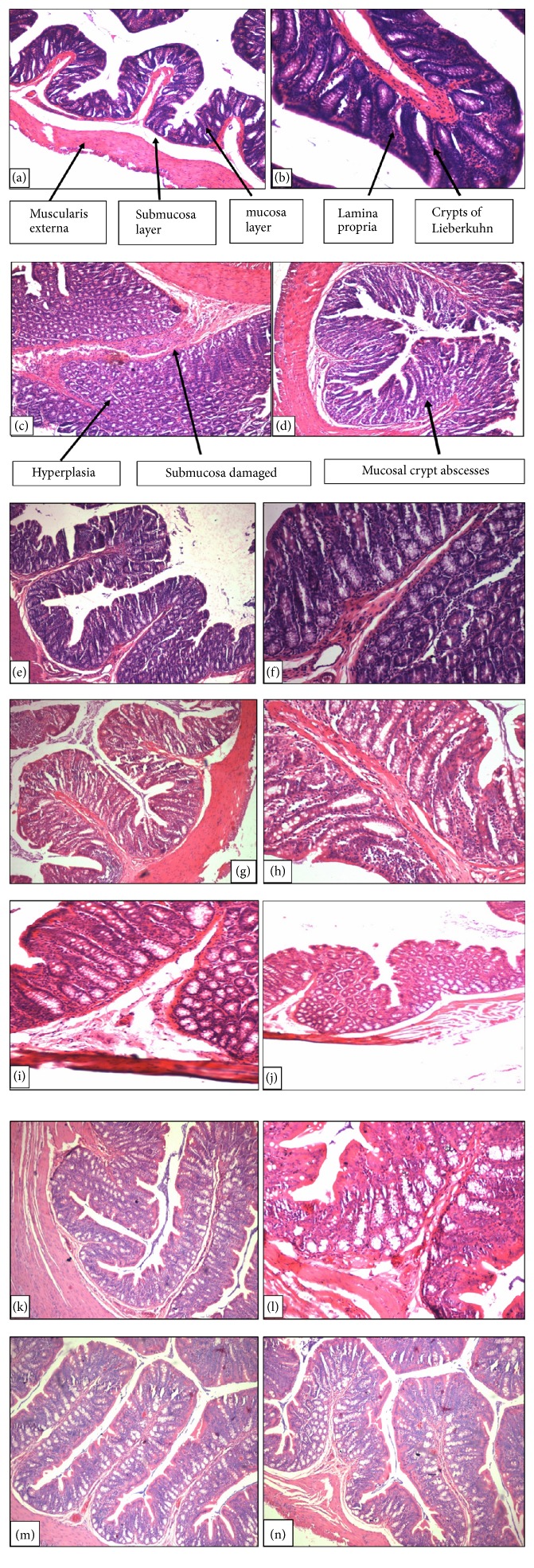
Histological examination of colon where 7(a) and 7(b) are control; 7(c) and 7(d) are DMH control; 7(e) and 7(f) are 5-fluorouracil; 7(g) and 7(h) are treatment group (100mg/kg); 7(i) and 7(j) are treatment group (200mg/kg); 7(k) and 7(l) are pretreatment group (100mg/kg); 7(m) and 7(n) are pretreatment group (200mg/kg).

*Histopathological Examination*. The histopathological examination of colon for different treatment groups is shown in Figure 7.

Figures 7(a), 7(c), 7(e), 7(g), 7(i), 7(k), and 7(m) are under 10X magnification and Figures 7(b), 7(d), 7(f), 7(h), 7(j), 7(l), and 7(n) are under 40X magnification. In the control group (Figures 7(a) and 7(b)), a regular arrangement of cells was observed showing normal muscularis externa, submucosa, mucosa, lamina propria, and crypts of Lieberkuhn. In DMH control group (Figures 7(c) and 7(d)), there is a hyperproliferation of cells with submucosa and mucosa being damaged. Additionally, abscess in crypt of Lieberkuhn, high aberrant crypt foci, and enlargement of nucleus and proliferation of cells have been reported. Less number of hyperproliferative cells and normal structures close to the control group have been shown in Figures 7(e) and 7(f) for 5-Fluorouracil group. Figures 7(g) and 7(h) showed less number of hyperproliferative cells, similar to control which is the treatment group at 100 mg/kg. In the treatment group (200mg/kg), as evident in Figures 7(i) and 7(j), histology of mucosa is near to normal still denoting a few hyperproliferative cells. Figures 7(k) and 7(l) are of pretreatment groups (100mg/kg), which indicates a lesser number of hyperproliferative cells compared to disease and control groups. The least number of hyperproliferative cells compared to disease and control was observed in pretreatment (200mg/kg) (Figures 7(m) and 7(n)).

## 4. Discussion

Cancer is a disease characterized by uncontrolled division and survival of abnormal cells. The abnormal growth that occurs in the colon or rectum is known as colorectal cancer. Colon cancer usually starts as noncancerous growth known as polyp on the inner wall of colon or rectum. This polyp grows slowly and develops into an adenomatous polyp or adenoma for a period of 10 to 20 years. Colon cancer is a multistage process, which involves initiation, promotion, and progression. DMH is a chemical carcinogen, which is well-established for inducing colon cancer* in vivo. *DMH when administered intraperitoneally is metabolized by liver to series of intermediates like azoxymethane, methylazoxymethanol acetate (MAMA), and methyl diazonium ions. The MAMA enters into the epithelial cells of colon and causes alkylation of DNA. The earliest hallmark for colon cancer is aberrant crypt foci (ACF). These are enlarged, aberrant, and elliptical shape crypts, which can be distinguished by comparing with control group. In our study, DMH administered animals showed ACF initiation and adenoma development which indicated not only the onset, but also the progression of colon cancer [16].

The small and large adenomas develop due to mutations in K-ras, hypo-Met-DNA [25]. These adenomas are further progressed into adenocarcinoma by mutations in p53 gene and mismatch repair mutations [26, 27].

The presence of flavonoids and triterpenoids could be one of the contributing factors for mechanism of anticancer action since one such flavonoid-cucurbitacin is known for its anticancer effect. The spleen and liver indices are important hallmarks in the detection of metastasis of colon cancer, splenomegaly, and hepatomegaly. However, there was no significant difference in the liver and spleen index of all treatment groups, which suggests that there is no damage to liver and spleen. In tumour tissue, the level of catalase was decreased. The enzyme catalase is mainly used by the cells to defend against hydrogen peroxide, which is generated by various reactions. Glutathione content was more in DMH control because of the oxidative stress involved in the process of tumour development. Polyunsaturated fatty acids present in cell membrane is oxidized by reactive oxygen species. The above reaction initiates lipid peroxidation, a chain reaction that produces free radicals and substances such as hydroperoxides, malondialdehyde, toxic aldehydes, and lipoperoxides. Malondialdehyde acts as signal transducers which induces cell proliferation and finally contributes to colon cancer. The initiation of colon cancer and its progression mainly depends upon reactive oxygen metabolites. Superoxide dismutase plays an important role in decreasing the free radicals in colon cancer. Pumpkin seed extract at a dose of 200 mg/kg as preventive measure decreased the colon length/weight ratio. Nitric oxide (NO) is a free radical which is responsible for cell signaling and cell damage. It reacts with superoxide (O^2-^) and forms peroxynitrite. This peroxynitrite interacts with DNA, proteins, and lipids and is one of the underlying mechanisms for cancer. In our study, nitrite levels were significantly enhanced in DMH group as compared to control group.

Previous reports state that 60% ethanolic extract of pumpkin seed suppressed colon cancer in vitro which was conducted on colon cancer cell lines. Crude pumpkin seed extract efficacy was tested on androgen sensitive (LNCap), androgen insensitive (DU-145), MCF-7 (breast cancer cell lines), and Caco-2 (colorectal adenocarcinoma cell lines). In Caco-2 cell lines, there was an inhibition of hyperplastic cells. Researchers have reported that the anticancer effect was not because of cucurbitacin, which is one of the active constituents in pumpkin seed extract. This particular evidence goes without fail, that there are other mechanisms responsible for anticancer activities. Further, isolation studies are required to elucidate the probable cause for anticancer effect [12].

The extent of damage to the colon by DMH is evaluated by increase in ACF count and polyp count. In DMH control, there are a high number of ACF, adenoma, and polyps. In the 5-Fluorouracil treatment, pumpkin seed extract showed good anticancer activities by maintaining the low ACF count, polyp count when compared to DMH control group. The treatment groups also showed significant increase in the levels of antioxidant enzymes when compared to DMH control. Pumpkin seed extract showed comparable or lower efficacy with standard 5-Fluorouracil treatment in this model with an apparent increase in endogenous antioxidant levels. Histopathological results showed abscess in crypt of Lieberkuhn, high aberrant crypt foci, and enlargement of nucleus and proliferation of cells in DMH group. A lesser number of aberrant crypt foci, hyperplastic cells, and no abscess in mucosal crypts were observed in treatment groups.

Cucurbitacins present in the pumpkin seed are responsible for inducing apoptosis and have the ability to modify genes, transcriptional activities, and also activation and inhibition of pro- or antiapoptotic proteins. Cucurbitacins mainly act by inhibition of JAK/STAT pathways and other mechanisms related to the antiapoptotic effect such as PARP cleavage, MAPK pathway, activating caspase-3, JAK3, and decreased pSTAT3 levels. They also decrease targets for STAT3such as Cyclin D3, Bcl-2, Mcl-1, and Bcl-xL, which play main role in the cell cycle control [28].

Based on our findings, which are comprised of less ACF count, increased antioxidant levels, and restored histopathological findings, we can conclude that PS treatment and pretreatment at 200 mg/kg showed better anticancer activity when compared to the lower dose. Pumpkin seed can be a promising candidate for treating colon cancer by normalizing levels of antioxidant enzymes, inhibiting hyperplastic cells, increasing the colon length/weight ratio, and decreasing ACF count.

## 5. Conclusion

To conclude, the present study provides evidence for the protective effects of pumpkin seeds in dimethylhydrazine (DMH) induced colon cancer. Its actions are related to attenuation of hyperplastic cells in colonic mucosa, modulation of antioxidants, and restoration of normal histological features. Further studies are warranted to decipher the probable mechanism by which pumpkin seeds exert anticancer effect. Nevertheless, consumption of seeds of pumpkin in dietary proportions would benefit people suffering from colon cancer rather than harming them.

## Figures and Tables

**Table 1:** Qualitative analysis of pumpkin seed extract.

**Phytoconstituents**	**Result**
Alkaloid	-ve
Carbohydrate	-ve
Flavonoids	+ve
Triterpenoids	+ve
Steroids	+ve
Tannins	+ve
Phytosterol	+ve
Saponins	+ve

**Table 2:** Effect of pumpkin seed extract on ACF count expressed in number of ACF/5cm^2^.

**Treatment**	**Number of ACF/5 cm** ^**2**^
Control	0
DMH control	151.83 ± 9.24^(a)^
Standard	66.16 ± 7.86^(a,b)^
Treatment – 100mg/kg	76.33 ± 22.83^(a,b)^
Treatment – 200mg/kg	54 ± 10.42^(a,b)^
Pretreatment-100mg/kg	67.83 ± 17.76^(a,b)^
Pretreatment-200mg/kg	51.83 ± 9.14^(a,b)^

All values are mean ± SEM of six samples; ^a^P<0.05 versus control; ^b^P<0.05 versus DMH control.

**Table 3:** Haematological parameters.

**Group**	**WBC**	%**Lymphocytes**	%**Granulocytes**	%**Monocytes**
**Control**	77.6±14.76	65.96±11.54	43.33±17.91	23.98±21.5
**DMH control**	80.2±9.96	65.7±11.01	40.53±17.02	27.05±20.77
**5-Flurouracil**	60.1±12.86^(a,b)^	59.63±5.23	28.08±3.708	12.28±2.811
**Treatment 100mg/kg**	47.91±5.89^(a,b)^	68.08±9.50	24.85±2.46	28.6±20.2^(a,b)^
**Treatment 200 mg/kg**	34.48±5.03^(a,b)^	46.03±5.19^(a)^	34.46±3.12^(a,b)^	15.18±3.85
**Pre-Treatment– 100mg/kg**	29.86±4.75^(a,b)^	41.03±11.71	48.66±11.8^(a,b)^	10.3±0.889
**Pre-Treatment 200 mg/kg**	7.61±6.17^(a,b)^	35.7±10.24^(a,b)^	54.8±12.28^(a,b)^	9.5±2.447

All values are mean ± SEM of six samples; ^a^P<0.05 versus control; ^b^P<0.05 versus DMH control.

**Table 4:** Effect of pumpkin seed extract on colon length/weight ratio.

**Treatment**	**Colon L/W ratio (cm/g)**
Control	10.76 ± 0.974
DMH control	7.945 ± 0.586^(a)^
Standard	9.929 ± 1.364
Treatment – 100mg/kg	8.715 ± 0.834
Treatment – 200mg/kg	8.234 ± 1.015
Pretreatment-100mg/kg	9.796 ± 0.676
Pretreatment-200mg/kg	10.559 ± 1.379^(b)^

All values are mean ± SEM of six samples; ^a^P<0.05 versus control; ^b^P<0.05 versus DMH control.

**Table 5:** Effect of PS on spleen index and liver index.

Treatment	Spleen index (mean ± SEM)	Liver index (mean ± SEM)
Control	0.0038 ± 0.0005	0.0237 ± 0.0041
DMH control	0.00514 ± 0.0004	0.0327 ± 0.0016
Standard	0.00452 ± 0.0005	0.0279 ± 0.002
Treatment – 100mg/kg	0.00418 ± 0.000387	0.0309 ± 0.0016
Treatment – 200mg/kg	0.008551 ± 0.0058	0.0567 ± 0.0283^(a)^
Pretreatment-100mg/kg	0.00538 ± 0.0007	0.0296 ± 0.0037
Pretreatment-200mg/kg	0.00558 ± 0.00026^(a)^	0.0350 ± 0.0024^(a)^

All values are mean ± SEM of six samples; ^a^P<0.05 versus control.

**Table 6:** Effect of pumpkin seed extract on catalase.

**Treatment**	**Catalase unit/mg of protein (mean ± SEM)**
Control	685.158 ± 101.94
DMH control	249.51 ± 38.96^(a)^
Standard	592.98 ± 109.30
Treatment – 100mg/kg	552.89 ± 110.88^(b)^
Treatment – 200mg/kg	573.97 ± 142.52^(b)^
Pretreatment-100mg/kg	684.77 ± 191.25^(b)^
Pretreatment-200mg/kg	463.642 ± 33.96

All values are mean ± SEM of six samples; ^a^P<0.05 versus control; ^b^P<0.05 versus DMH control.

**Table 7:** Effect of pumpkin seed extract on GSH.

**Treatment**	**GSH ** ***μ*** **M/mg of protein (mean ± SEM)**
Control	47.252 ± 6.877
DMH control	25.042 ± 1.199^(a)^
Standard	36.935 ± 4.351^(b)^
Treatment – 100mg/kg	41.313 ± 4.186^(b)^
Treatment – 200mg/kg	36.185 ± 4.086^(b)^
Pretreatment-100mg/kg	38.396 ± 3.34^(b)^
Pretreatment-200mg/kg	40.663 ± 2.880^(b)^

All values are mean ± SEM of six samples; ^a^P<0.05 versus control; ^b^P<0.05 versus DMH control.

**Table 8:** Effect of pumpkin seed extract on lipid peroxidation/MDA content.

**Treatment**	**Nano moles of MDA/mg of protein (mean ± SEM)**
Control	374.029 ± 61.11
DMH control	260.79 ± 26.233
Standard	263.386 ± 82.49^(a)^
Treatment – 100mg/kg	188.74 ± 51.20^(a)^
Treatment – 200mg/kg	160.73 ± 53.52^(a,b)^
Pretreatment-100mg/kg	301.255 ± 108.15^(a)^
Pretreatment-200mg/kg	166.745 ± 37.523^(a)^

All values are mean ± SEM of six samples; ^a^P<0.05 versus control; ^b^P<0.05 versus DMH control.

**Table 9:** Effect of pumpkin seed extract on SOD.

**Treatment**	**SOD /mg of protein** ** (mean ± SEM)**
Control	2.427 ± 0.319
DMH control	0.3008 ± 0.0356^(a)^
5-FU (standard)	1.4264 ± 0.6080
Treatment – 100mg/kg	2.0770 ± 0.3134^(b)^
Treatment – 200mg/kg	2.561 ± 0.4739^(b)^
Pretreatment-100mg/kg	2.3046 ± 0.8133^(b)^
Pretreatment-200mg/kg	1.8991 ± 0.7544^(b)^

All values are mean ± SEM of six samples; ^a^P<0.05 versus control; ^a^P<0.05 versus DMH control.

**Table 10:** Effect of pumpkin seed extract on nitrite content.

Treatment	*μ*M /mg of protein (mean ± SEM)
Control	6.874 ± 2.356
DMH control	8.506 ±3.866
5-FU (Standard)	5.867 ± 1.739^(b)^
Treatment – 100mg/kg	6.461 ± 1.093
Treatment – 200mg/kg	5.203 ± 0.852^(b)^
Pretreatment-100mg/kg	6.971 ± 0.9477
Pretreatment-200mg/kg	7.199 ± 1.7439

All values are mean ± SEM of six samples; ^b^P<0.05 versus DMH control.

## Data Availability

The data used to support the findings of this study are included within the article.
